# Editorial: Fluorescent Carbon-Based Nanostructures for Bioimaging Applications

**DOI:** 10.3389/fchem.2020.587918

**Published:** 2020-09-15

**Authors:** Timur Sh. Atabaev, Dong-Wook Han

**Affiliations:** ^1^Department of Chemistry, Nazarbayev University, Nur-Sultan, Kazakhstan; ^2^Department of Cogno-Mechatronics Engineering, College of Nanoscience and Nanotechnology, Pusan National University, Busan, South Korea

**Keywords:** carbon quantum dots, graphene quantum dots, fluorescence, bioimaging, upconversion, MRI

Recently discovered carbon quantum dots (CQDs) and graphene quantum dots (GQDs) with excellent optical properties sparked considerable interest in the scientific community. First of all, these CQDs and GQDs can be conveniently prepared using green protocols from virtually any carbon-rich media making them more attractive compared to other optical materials. Second, the emission spectrum of CQDs and GQDs can be tuned by controlling the reaction parameters or by incorporation of dopant ions. Finally, low-cost, non-toxic, inert, and photostable CQDs and GQDs already exhibited promising results in areas such as bioimaging, sensing and detection, photocatalysis, energy, and security printing.

In this special issue, we placed a special focus on the synthesis of novel fluorescent carbon-based nanomaterials for bioimaging purposes. In addition to that, several works also covered other interesting areas such as sensing and detection. In total, one review article and four research articles were published in this special issue after a rigorous peer-review process.

A review article titled “Recent Advances on Graphene Quantum Dots for Bioimaging Applications” prepared by Younis et al. explicitly covers top-down and bottom-up strategies for the synthesis of GQDs. In addition, the authors covered fluorescence, two-photon, magnetic resonance, and dual-mode bioimaging applications of GQDs with selected examples from the scientific literature. Finally, toxicity issues, current challenges, and future prospects were deliberated.

Liu et al. prepared a multifunctional nanocomposite comprising of Fe_3_O_4_, upconversion nanoparticles, and hollow carbon sphere. Thanks to the presence of Fe_3_O_4_, obtained nanocomposite can be utilized as an MRI contrast agent with a high r_2_ value of 57.7 mM^−1^ s^−1^. Under 980 nm excitation, upconversion nanoparticles can generate green and red fluorescent signals suitable for deep and safe NIR-excitable bioimaging. Furthermore, the carbon sphere can convert the absorbed NIR radiation into heat making this nanocomposite suitable for photothermal therapy. Excellent biocompatibility and multimodality of prepared nanocomposite can be useful for the development of future contrast agents.

Zhang et al. reported the synthesis of novel CQDs using the Rosa roxburghii biomass precursor. According to their results, CQDs with a mean size of 2.5 nm and quantum yield of 24.8% can be obtained. These CQDs showed good biocompatibility and were suitable for fluorescence imaging of Hep3B cells. Moreover, these CQDs can be also used for o-nitrophenol detection in the linear range of 0.08–40 μmol/L with a limit of detection of 15.2 nmol/L.

The microwave-assisted method was used by Chen et al. for the synthesis of fluorescent CQDs with an average size of 4.5 nm and quantum yield of 12.45%. The authors demonstrated that prepared CQDs can be used for selective Fe^3+^ detection in the range of 8–80 μM with a limit of detection of 3.8 μM. Later on, the authors demonstrated that these CQDs can serve as a fluorescent label for bioimaging purposes and for intracellular Fe^3+^ detection inside fungal cells.

Niu et al. utilized fluorescent GQDs with an average diameter of 5 nm for the detection of sunset yellow food additive using the electrochemiluminescence (ECL) method. Under optimized conditions, GCDs based sensor is suitable for sensitive detection of sunset yellow in the range of 0.0025–25 μM with a limit of detection of 7.6 nM. A proposed method can serve as a basis for the development of low-cost and rapid screening methods of some food additives.

Thanks to their unique fluorescent properties, CQDs and GQDs have been extensively applied for bioimaging, sensing, catalytic, and solar light conversion purposes. In general, synthesis protocols, size and shape control, tuning of fluorescence emission has been advanced significantly in recent years. On the other hand, some important questions still need to be investigated. Among them, the origin of excitation-dependent emission, the position of dopant ions and their role in fluorescence emission, and the mechanism of upconversion emission. Therefore, this special issue collected recent promising studies that substantially improved the scientific knowledge and partially filled the existing gaps. On the other hand, some unresolved questions still remain, especially, the fundamental understanding of the fluorescence processes in CQDs and GQDs. We hope, that these important questions will be addressed in the near future.

It was a great pleasure for us to edit such an exciting special issue for Frontiers in Chemistry journal. The guest editors hope that this collection of articles will gain considerable attention from the scientific community.

## Author Contributions

All authors listed have made a substantial, direct and intellectual contribution to the work, and approved it for publication.

## Conflict of Interest

The authors declare that the research was conducted in the absence of any commercial or financial relationships that could be construed as a potential conflict of interest.

